# Tendon-Targeted Knockout of Collagen XII Disrupts Murine Achilles Tendon Structure, Function, and Gene Expression at Later Stages of Postnatal Development

**DOI:** 10.1007/s10439-026-04125-6

**Published:** 2026-03-31

**Authors:** Michael S. DiStefano, Rebecca L. Betts, Courtney A. Nuss, Srish S. Chenna, Louis J. Soslowsky

**Affiliations:** 1 McKay Orthopaedic Laboratory, University of Pennsylvania, 307A Stemmler Hall, 3450 Hamilton Walk, Philadelphia, PA 19104-6081, USA; 2 Department of Bioengineering, University of Pennsylvania, Philadelphia, PA, USA

**Keywords:** Achilles tendon, Collagen XII, Tendon development, Structure function, Gene expression

## Abstract

Tendons rely on hierarchical extracellular matrix organization for proper growth and mechanical function, but the tendon-specific role of collagen XII in this process remains poorly defined. The purpose of this study was to determine how tendon-targeted loss of collagen XII influences Achilles tendon structure, mechanics, and gene expression across key stages of postnatal development. Using tendon-targeted Scx-Cre;*Col12a1*^flox/flox^ knockout mice, we evaluated Achilles tendons at postnatal days 10, 30, and 60 with histological, ultrastructural, biomechanical, and compositional approaches. Mechanical testing revealed progressive deficits in knockout tendons, including reduced maximum load, stiffness, and modulus at P30 and P60, as well as altered viscoelastic properties across all timepoints. Structural analyses demonstrated increased tendon length across all timepoints and greater cell density, rounder nuclei, impaired collagen fiber realignment, and smaller fibril diameters with disrupted maturation, with more notable changes at later postnatal developmental timepoints. Gene expression profiling revealed early downregulation of matrix and signaling genes at P10, followed by upregulation of matrix remodeling and matricellular genes at P30 and P60, consistent with a persistent, maladaptive remodeling response. Together, these findings establish collagen XII as a critical regulator of hierarchical tendon development, integrating fibrillogenesis, cell morphology, mechanical function, and gene expression to ensure proper tendon development.

## Introduction

Tendons possess a highly structured, hierarchical organization that plays a crucial role in determining their mechanical performance. Yet, restoring this complex structure after injury remains a major clinical challenge. While collagen I is the dominant contributor to the tendon’s tensile strength, lesser-known fibrillar and non-fibrillar collagens, such as collagen XII, are also essential in shaping tendon architecture and function. Collagen XII belongs to the FACIT (fibril-associated collagens with interrupted triple helices) family. It is a homotrimer, composed of three identical chains that contain alternating collagenous and non-collagenous domains. Through its collagenous domains, collagen XII attaches to collagen I fibrils, while its extended third non-collagenous domain projects into the interfibrillar space. The protruding domains interact with matrix components like tenascin-X and decorin, forming flexible bridges that help organize and stabilize fibril alignment [1, 2]. Mutations in the *Col12a1* gene are linked to myopathic Ehlers-Danlos syndrome (EDS), a condition that combines features of both muscle and connective tissue disorders [3]. Affected individuals often experience distal joint hypermobility, proximal joint contractures, muscle weakness, abnormal scarring, and absent deep tendon reflexes [3]. These clinical manifestations suggest that collagen XII is critical for tendon health, although its precise role in regulating tendon structure and mechanical function remains incompletely defined.

Using a *Col12a1* global knockout mouse model, we previously demonstrated that flexor digitorum longus (FDL) tendons in these mice possessed greater cross-sectional area (CSA) and stiffness, but did not exhibit a difference in elastic modulus relative to wild-type controls [4]. FDL tendons also demonstrated abnormalities in fiber and fibril structure, where postnatal day 30 (P30) global knockout mice exhibited poorly defined fiber domains, disrupted organization, and truncated cellular processes. At the nanoscale level, fibril packing was reduced with the presence of irregularly shaped fibrils in knockout mice. Tendon findings in the global *Col12a1* knockout model support important roles for collagen XII in regulating fibrillogenesis, fiber and fibril organization, and cell shape and organization. However, a key limitation of this global knockout model is the potential confounding effects of collagen XII knockout in other tissues, such as muscle and bone, and the specific role of collagen XII in tendon has not been elucidated.

To specifically isolate the role of collagen XII in tendon, we developed a conditional *Col12a1*-null (*Col12a1*^flox/flox^) mouse model using a promoter-driven knockout embryonic 15 stem cell line [5]. Notably, this model results in no expression of the short and long *Col12a1* transcripts, and because collagen XII is a homotrimer, all possible isoforms of collagen XII are knocked out. These mice were then crossed with scleraxis (Scx)-Cre mice to obtain tendon-targeted homozygous collagen XII knockout (Scx-Cre; *Col12a1*^flox/flox^; KO) mice. Grip strength testing in KO mice revealed deficits consistent with those previously reported in the global KO model. Mechanical testing of FDL tendons at P60 further demonstrated impaired function, as KO tendons displayed decreased stiffness and modulus relative to wild-type controls [5]. Taken together, findings from both global and tendon-specific collagen XII knockout models establish collagen XII as a key regulator of tendon function. However, a comprehensive understanding of the role of collagen XII on the structural, functional, and compositional properties of tendon throughout postnatal development is warranted.

Therefore, the objective of this study was to define the role of collagen XII in regulating hierarchical assembly of tendon required for function during postnatal development. We hypothesized that tendon-targeted knockout of collagen XII would disrupt tendon homeostasis, leading to altered Achilles tendon mechanics, morphology, and gene expression, with more notable differences at later postnatal developmental timepoints when ECM establishment and maturation are occurring. To test this hypothesis, tendon-targeted Scx-Cre collagen XII knockout mice were used to knockout collagen XII throughout tendon development and tendon structure, function, and gene expression were evaluated at P10, P30, and P60.

## Methods

### Animal Use and Study Design

This study was approved by the University of Pennsylvania Institutional Animal Care and Use Committee (IACUC) and carried out in strict accordance with guidelines established in the *Guide for the Care and Use of Laboratory Animals*. C57BL/6 male and female mice were housed in an AAALAC accredited facility that maintained 12-h light/dark cycles, temperatures between 20 and 26 °C and humidity between 30 and 70%. The role of collagen XII in regulating Achilles tendon structure, mechanical function, and gene expression was evaluated using tendon-specific knockout mice (Scx-Cre;*Col12a1*^flox/flox^; KO) and compared to Cre-negative littermate controls (WT). By targeting the deletion to scleraxis-expressing cells, the model minimizes off-target effects and more specifically isolates collagen XII’s function within tendons. The cumulative effect of collagen XII deficiency on cellular structure, mechanics, and gene expression was assessed in Achilles tendons from P10, 30, and 60 mice. P10 represents a developmental transition from cell proliferation to matrix deposition and assembly, while by P30, tendon hierarchical structure is formed, although the mice are not yet sexually mature. At P60, the tendon hierarchical structure is fully established and the mice are sexually mature [6]. This study utilized a multidisciplinary approach to assess tendon structural, functional, and compositional changes with tendon-targeted collagen XII knockout. Functional properties of the matrix were tested using quasi-static and viscoelastic mechanical assays (*n* = 10 per group). Matrix structure was examined using paraffin histology (*n* = 5–8 per group) to assess Achilles tendon length, cell density, and nuclear aspect ratio and TEM to assess fibril morphology (*n* = 8 per group). Composition was evaluated with gene expression profiling of selected targets (*n* = 10 per group).

Tendon-specific collagen XII knockout mice were generated using the scleraxis (Scx)-Cre;*Col12a1*^flox/flox^ model. Scx-Cre mice were bred to homozygous *Col12a1*^flox/flox^ mice, with the final breeding scheme involving female Scx-Cre +;*Col12a1*^flox/flox^ crossed with male *Col12a1*^flox/flox^. Only female Cre + mice were used for breeding to avoid potential Cre-mediated recombination in the sperm of male mice. Cre-negative littermates served as controls.

### Biomechanical Testing and Collagen Fiber Realignment Analysis

Achilles tendons were collected from P10, P30, and P60 wild-type (WT) and knockout (KO) mice. CSA measurements were obtained using a custom-built laser device, as described.[7] Mechanical testing was conducted on an Instron 5848 system (Instron 5848; Instron, Norwood, MA) using a viscoelastic protocol. This included a preconditioning phase (10 cycles of 0.5% strain amplitude oscillations at 1 Hz centered around 1% strain), followed by 3 stress relaxation tests (3, 5, and 7% strain, applied rapidly and held for 5 minutes), a dynamic frequency sweep (10 cycles at 0.0125% strain amplitude across 0.1, 1, 5, and 10 Hz), and finally a quasi-static ramp to failure at a rate of 0.1% strain per second [8, 9]. Structural, material, and viscoelastic material properties were calculated using the CSA values and load–displacement data, analyzed with a custom script (MATLAB; Natick, MA) [8]. Collagen fiber realignment was also quantified from the ramp-to-failure images using cross-polarization imaging [10]. Quantified regional fiber realignment data, represented as circular variance, were normalized to the first discrete data point at 0% strain and interpolated in MATLAB using a polynomial fit as a function of strain from the load–displacement data.

### Cell Density and Nuclear Aspect Ratio

At the time of tissue collection, ankles from postnatal day 10, 30, and 60 mice were dissected, positioned at a 90-degree angle in histology cassettes, and fixed in 10% neutral buffered formalin for 24 h. Decalcification was carried out using a solution of 0.5-M EDTA (Thermo, Waltham, MA) for 7 to 10 d, with the solution refreshed every 2–3 d. Tissues were then dehydrated through a graded ethanol series, infiltrated with paraffin, and embedded [11]. Sagittal sections of the Achilles tendon were cut at 7 μm thickness, stained with 0.1% toluidine blue and DRAQ5 (Thermo, Waltham, MA), and imaged at 10X magnification using a ZEISS Axioscan 7. Tendon length was measured in ImageJ. Regions of interest (P10: 150×500 μm; P30 and P60: 300 × 800 μm) from the tendon midsubstance were used to quantify cell density and nuclear aspect ratio in CellProfiler.

### Collagen Fibril Morphology

Achilles tendons from P10, P30, and P60 mice were dissected and fixed in Karnovsky’s fixative (4% paraformaldehyde, 2.5% glutaraldehyde, 0.1-M sodium cacodylate, 8.0 mM calcium chloride), followed by post-fixation in 1% osmium tetroxide, dehydration through an ethanol series, and embedding in Epon, as described [12, 13]. Transverse ultrathin sections (60–80 nm thick) were cut using an ultramicrotome and post-stained with UranyLess (EMS 22409) and 1% phosphotungstic acid. Sections were carefully collected from the midsubstance region of the Achilles tendon to ensure consistency across samples between genotypes at each developmental timepoint [11, 14]. Collagen fibril cross-sections were imaged at 60,000x magnification using a JEOL 1010 transmission electron microscope. At each developmental timepoint, fibril diameters were measured using a custom MATLAB script [8] and compared across genotype.

### Gene Expression Analysis

Achilles tendons were dissected from the calcaneal insertion to just below the myotendinous junction to include the full length of the tendon, then preserved in RNAlater. For RNA extraction, tendons were mechanically homogenized using a pestle in a phenol-based lysis buffer, TRIzol (Thermo, Waltham, MA), followed by chloroform-based phase separation. RNA was then isolated following the manufacturer’s instructions (Direct-zol RNA Microprep, Zymo, Irvine, CA). Extracted RNA was reverse-transcribed into cDNA (High-Capacity cDNA RT, Thermo, Waltham, MA) and pre-amplified for 15 cycles targeting 94 pre-selected probes (TaqMan, Thermo, Waltham, MA). Highly expressed genes, *Rn18s* and *Col1a1*, were excluded from pre-amplification. The resulting cDNA was loaded onto a 96.96 Dynamic Array IFC (BMKM-96.96, Standard BioTools, South San Francisco, CA). The target gene panel included collagens, non-collagenous matrix proteins, matrix remodeling enzymes, markers for cell–cell and cell–matrix interactions, signaling molecules, and cell identity markers (Supplemental Table S1). Cycle threshold (Ct) values were evaluated for each gene, including for the housekeeper genes *Rn18s*, *Abl1*, and *Rps17*. *Δ*Ct values were computed by subtracting gene Ct values from the average housekeeper Ct value for that sample.

### Statistics

Statistical analyses were performed in GraphPad Prism and R. Analyses were Student’s t-tests (statistical significance was set at *p* < 0.05) comparing WT and KO groups at each timepoint unless otherwise noted. Data distributions were checked for statistical outliers (1.5*IQR was decided *a priori*) and tested for normality (Shapiro–Wilk’s normality tests) prior to subsequent statistical analysis. WT and KO fibril diameter distributions from transmission electron microscopy were compared using Kolmogorov–Smirnov tests. Principal component analysis (PCA) was conducted on *Δ*Ct values for all samples. Volcano plots were generated to compare expression of target genes between genotypes, with statistical significance determined using Student’s *t* tests. Genes with both significant *p* values (*p* < 0.05) and log₂ fold changes > 0.5 or < −0.5 were identified as differentially upregulated or downregulated, respectively. Sample sizes for mechanical testing, histological analyses, ultrastructural assessment, and gene expression profiling were selected based on effect sizes and variability reported in prior murine tendon development and knockout studies [4, 11, 14, 15]. Mid-study power analyses were performed in MATLAB using observed variance from primary outcome measures to confirm that group sizes achieved at least 80% power.

## Results

### Validation of *Col12a1* Knockout Mouse Model

**Fig. 1:**
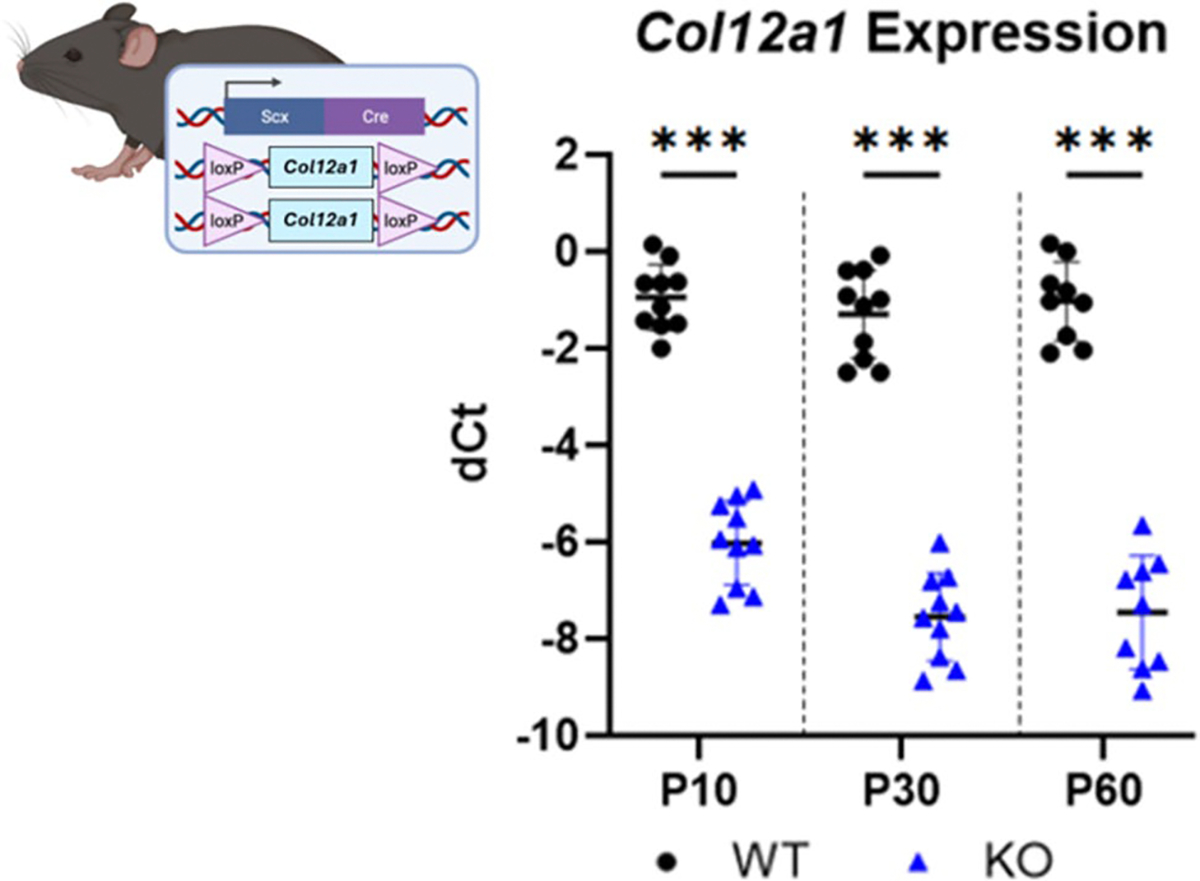
*Col12a1* expression was significantly reduced in KO Achilles tendons relative to WT control Achilles tendons across all timepoints. Data presented as mean ± standard deviation (*** *p* ≤ 0.001)

To examine how collagen XII influences tendon structure, function, and gene expression during postnatal development, Achilles tendons from tendon-targeted *Scx*-Cre;*Col12a1*^flox/flox^ mice were analyzed at postnatal days 10, 30, and 60. As expected and in line with earlier findings, *Col12a1* expression was markedly reduced in KO Achilles tendons across all timepoints (Fig. 1).

### Collagen XII Regulates Tendon Mechanical Function and Collagen Fiber Realignment

**Fig. 2:**
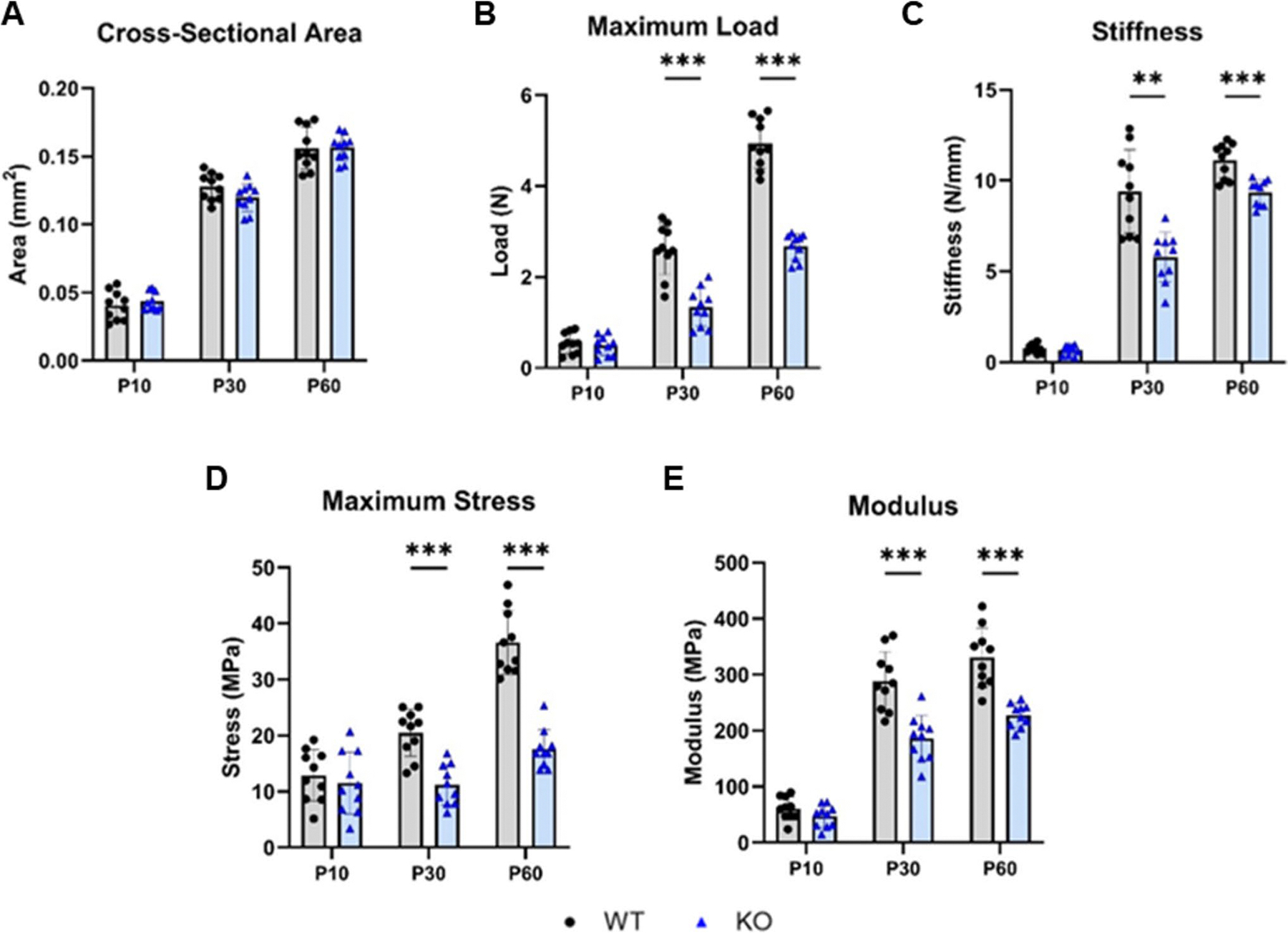
Tendon-targeted collagen XII KO did not result in any changes in **A** cross-sectional area relative to WT controls across all timepoints. However, KO tendons demonstrated reduced structural properties within **B** maximum load and **C** stiffness at P30 and P60, but not at P10. KO tendons exhibited reduced material properties **D** maximum stress and **E** modulus at later developmental timepoints, P30 and P60, but not at P10. Data presented as mean ± standard deviation (**p ≤ 0.01, *** *p* ≤ 0.001)

**Fig. 3:**
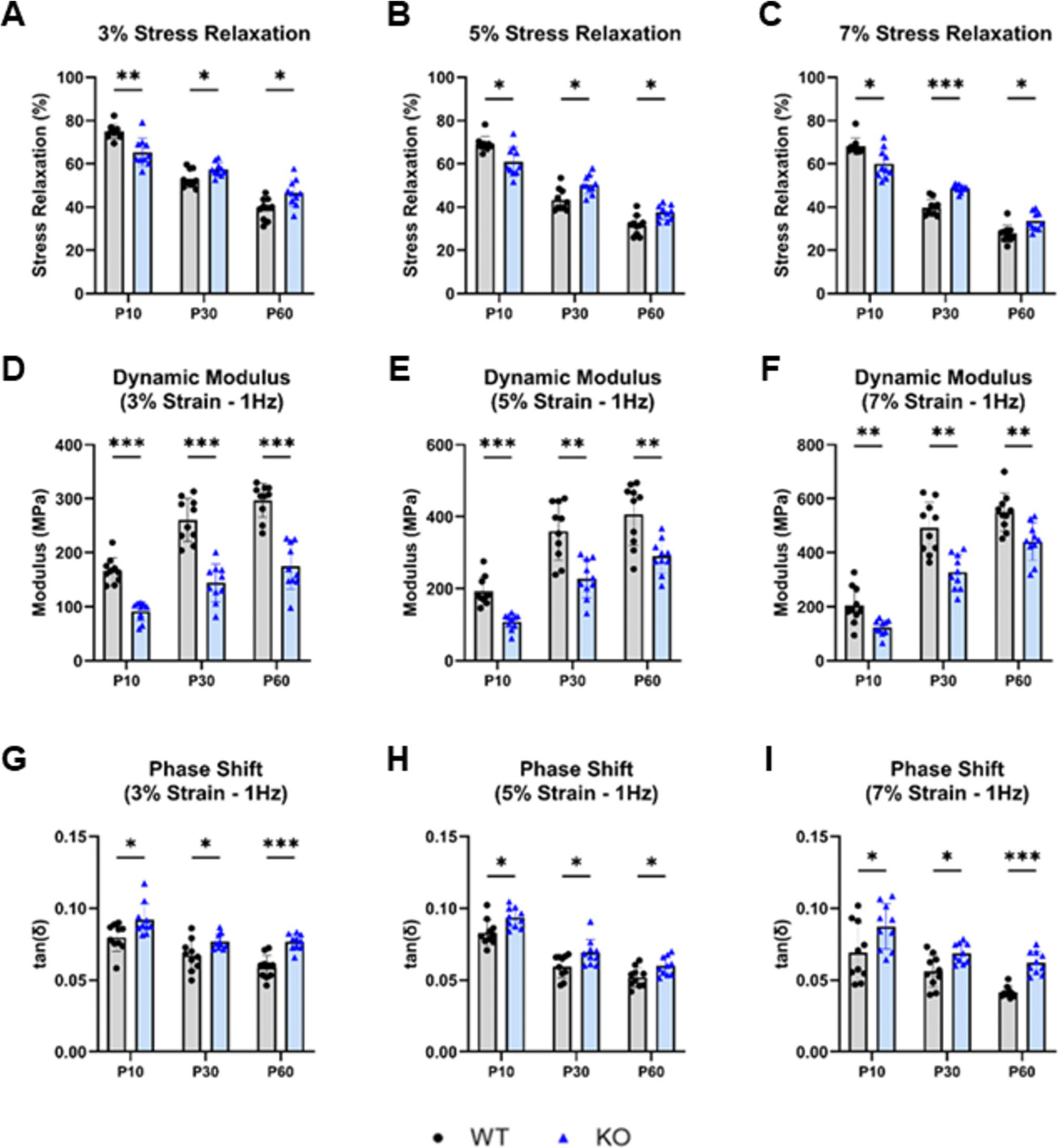
KO tendons demonstrated reduced stress relaxation relative to WT controls at P10 and increased stress relaxation relative to WT controls at P30 and P60 at **A** 3%, **B** 5%, and **C** 7% strain. (**D**–**F**) KO tendons demonstrated reduced dynamic modulus relative to WT controls across all timepoints and strain levels at 1 Hz. (G-I) KO tendons also exhibited increased phase shift at P10 across all strain levels at 1 Hz and decreased phase shift at P30 and P60 across all strain levels at 1 Hz relative to WT controls. Data presented as mean ± standard deviation (*p ≤ 0.05, **p ≤ 0.01, *** *p* ≤ 0.001)

To understand the role of tendon-targeted knockout of collagen XII on postnatal development tendon function, viscoelastic mechanical testing was performed at P10, P30, and P60 to assess structural and material elastic mechanical properties as well as viscoelastic mechanical properties. KO tendons demonstrated no changes in CSA (Fig. 2A) across all timepoints relative to WT control tendons. KO tendons exhibited reduced structural properties maximum load (Fig. 2B) and stiffness (Fig. 2C) compared to WT tendons at P30 and P60, but not at P10. Similarly, KO tendons demonstrated reductions in material properties maximum stress (Fig. 2D) and modulus (Fig. 2E) at P30 and P60, but not at P10. For viscoelastic properties, KO tendons demonstrated reduced stress relaxation relative to WT tendons at P10 and increased stress relaxation relative to WT tendons at P30 and P60 at 3% (Fig. 3A), 5% (Fig. 3B), and 7% (Fig. 3C) strain. Additionally, KO tendons demonstrated reduced dynamic modulus relative to WT tendons at 3% (Fig. 3D), 5% (Fig. 3E), and 7% (Fig. 3F) strain at 1 Hz at P10, P30, and P60. Phase shift was greater in KO tendons compared with WT tendons at P10, P30, and P60 at 3% (Fig. 3G), 5% (Fig. 3H), and 7% (Fig. 3I) strain at 1 Hz. Changes to dynamic modulus and phase shift at 0.1, 5, and 10 Hz were consistent with those observed at 1 Hz.

**Fig. 4:**
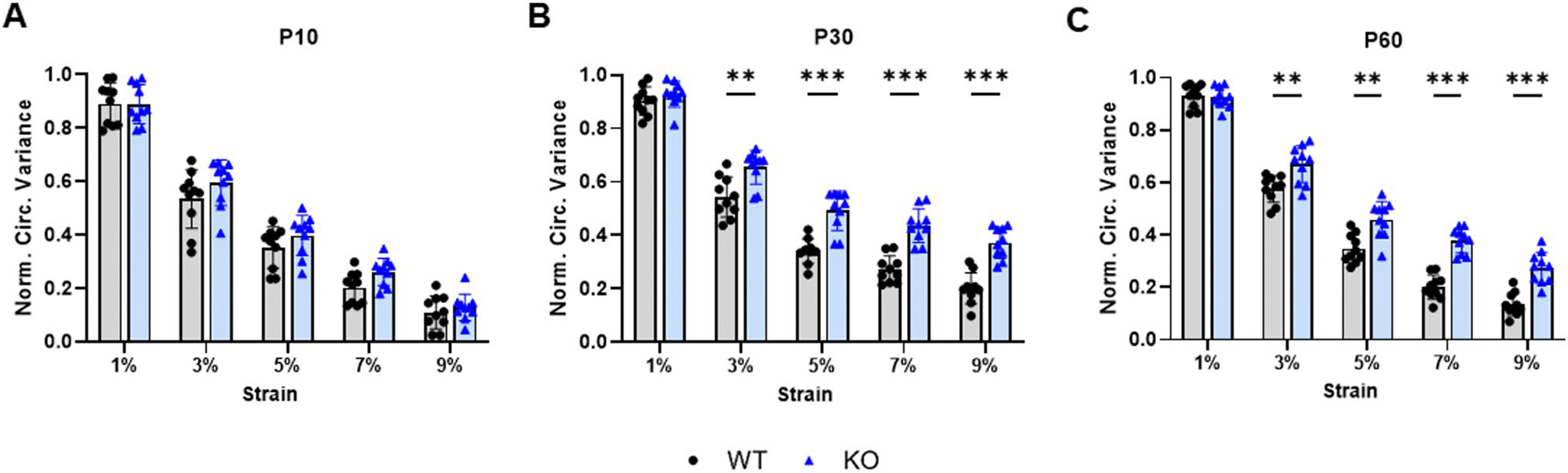
No differences were observed in collagen fiber realignment between WT and KO tendons at **A** P10. However, KO tendons demonstrated increased normalized circular variance, indicative of reduced collagen fiber realignment, at **B** P30 and **C** P60 from 3–9% strain, encompassing the toe and linear regions of these tendons. Data presented as mean ± standard deviation (**p ≤ 0.01, *** *p* ≤ 0.001)

In addition to mechanical differences observed at later postnatal developmental timepoints, KO tendons also demonstrated changes in collagen fiber realignment. Specifically, although no noticeable differences in normalized circular variance were observed at P10 (Fig. 4A), KO tendons demonstrated more notable increases in normalized circular variance, indicating reduced collagen fiber realignment, relative to WT tendons at P30 (Fig. 4B) and P60 (Fig. 4C). These differences are most evident from 3 to 9% strain, which encompass the toe and linear regions of these tendons and where realignment behavior of the collagen fibers during the quasi-static ramp-to-failure is occurring.

### Collagen XII Regulates Achilles Tendon Cell Density, Cell Shape, and Tendon Length

**Fig. 5:**
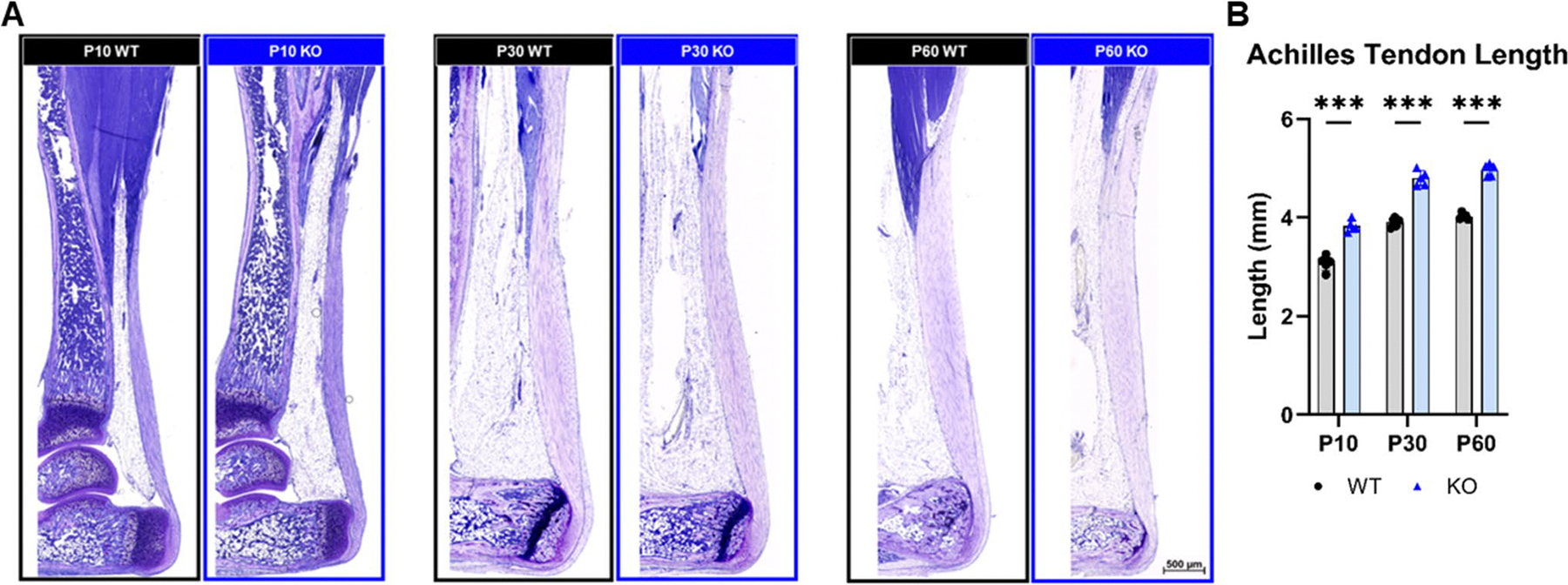
**A** Representative toluidine blue-stained sagittal histology sections for P10, P30, and P60 WT and KO Achilles tendons. **B** KO Achilles tendons demonstrated increased length relative to WT Achilles tendons at all timepoints. Data presented as mean ± standard deviation (***p ≤ 0.001)

**Fig. 6:**
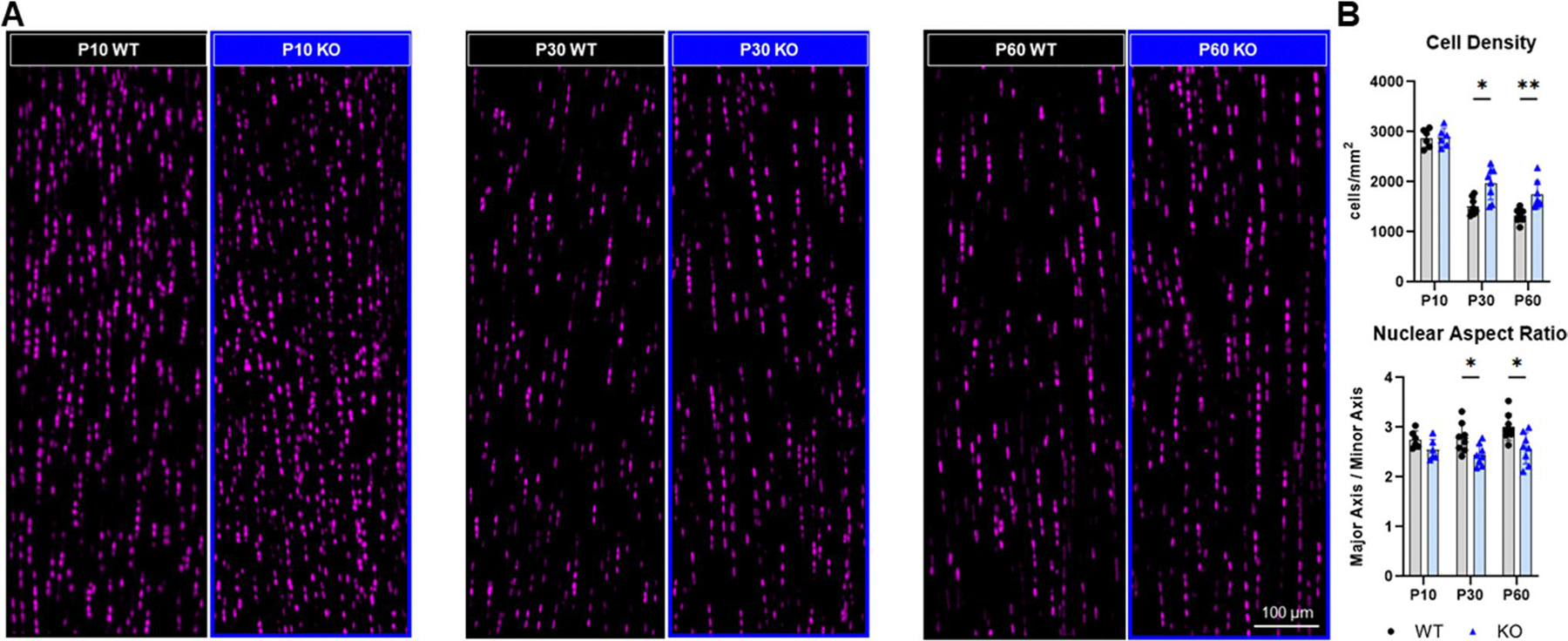
**A** Achilles tendons were stained with DRAQ5 to visualize nuclei for assessment of cell density and nuclear aspect ratio. **B** KO as tendons demonstrated increased cell density and decreased nuclear aspect ratio, indicative of more rounded cells, at P30 and P60. No changes in these parameters were observed at P10. Data presented mean ± standard deviation (*p ≤ 0.05, **p ≤ 0.01)

We evaluated Achilles tendon cellular and morphological changes with tendon-targeted collagen XII knockout using paraffin histology. Tendons were stained with toluidine blue to evaluate length changes during postnatal development (Fig. 5A). KO tendons exhibited greater length across all postnatal developmental timepoints (Fig. 5B). Achilles tendons were also stained with DRAQ5 to visualize nuclei for assessment of cell density and nuclear aspect ratio (Fig. 6A). KO tendons demonstrated increased cell density and decreased nuclear aspect ratio, indicative of more rounded cells, at P30 and P60. No changes in these parameters were observed at P10 (Fig. 6B).

### Collagen XII Knockout Alters Fibril Structure

**Fig. 7:**
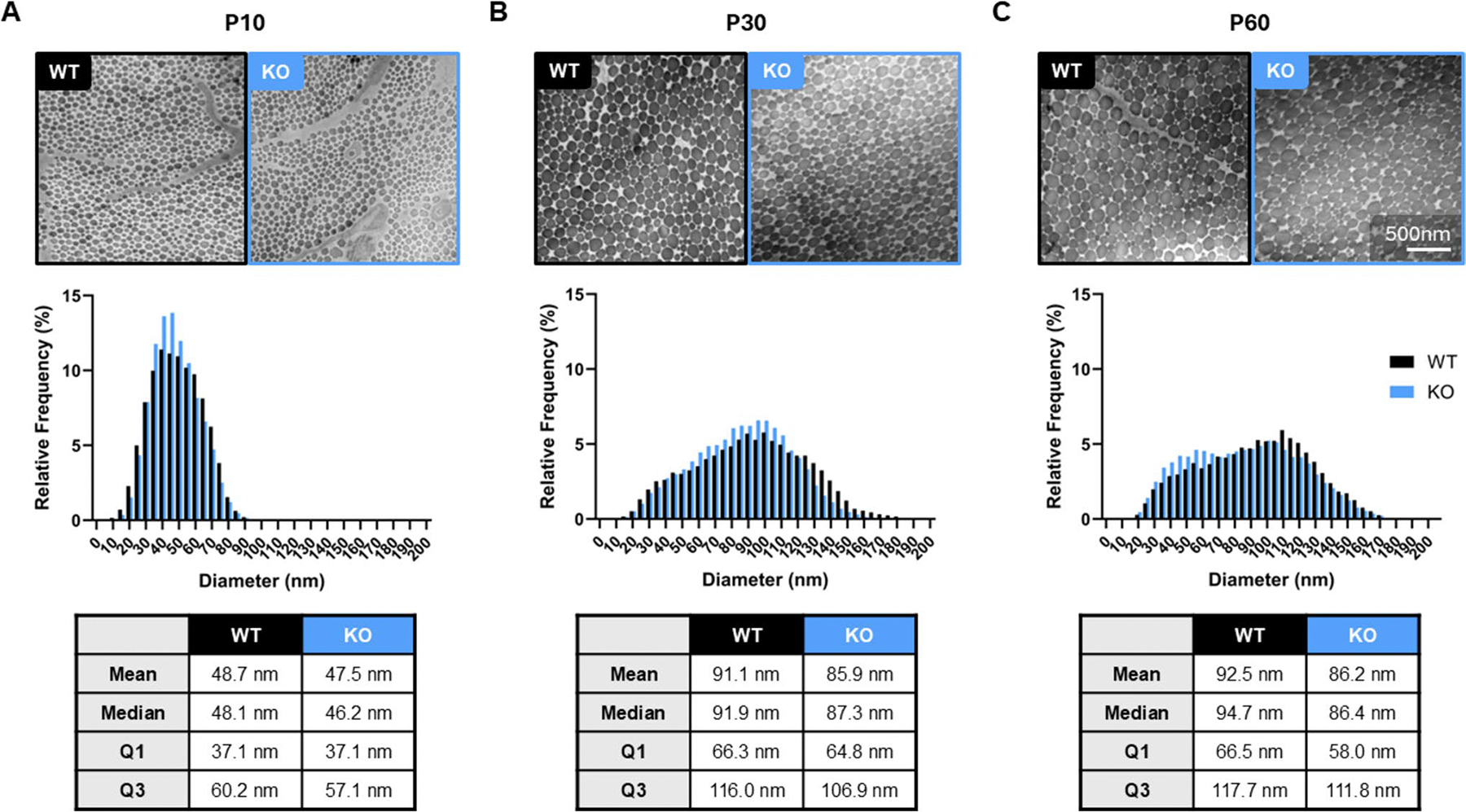
**A** The fibril diameter distributions at P10 exhibited minor differences between WT and KO tendons. **B** At P30, there was a slight shift toward larger diameter fibrils in WT tendons relative to KO tendons. **C** At P60, KO tendons demonstrated a more noticeable shift toward smaller diameter fibrils. Fibril diameter distributions were compared between WT and KO groups using Kolmogorov–Smirnov tests, which demonstrated statistical significance (*p* ≤ 0.001) between groups at all postnatal developmental timepoints

To investigate altered ECM deposition in KO tendons throughout postnatal development, we performed transmission electron microscopy on transverse sections of P10, P30, and P60 Achilles tendons. Using these transverse images, we segmented the deposited collagen fibril cross-sections from the surrounding interfibrillar matrix and compared fibril diameter distributions between WT and KO tendons. In P10 Achilles tendons, subtle, yet statistically significant (*p* < 0.001) differences were observed between WT and KO fibril diameter distributions. (Fig. 7A). At P30, when the tendon matrix is established, the distributions of collagen fibrils in KO tendons were shifted toward smaller diameter fibrils (*p* < 0.001), which is indicative of an overall less mature tendon ECM (Fig. 7B). At P60, when the tendon ECM has matured, a more noticeable shift was evident with an apparent peak in the distribution of smaller diameter fibrils in KO tendons (p < 0.001), indicating that the difference in deposited ECM continues at this later developmental timepoint (Fig. 7C).

### Collagen XII Knockout Alters Tendon Gene Expression Profiles

**Fig. 8:**
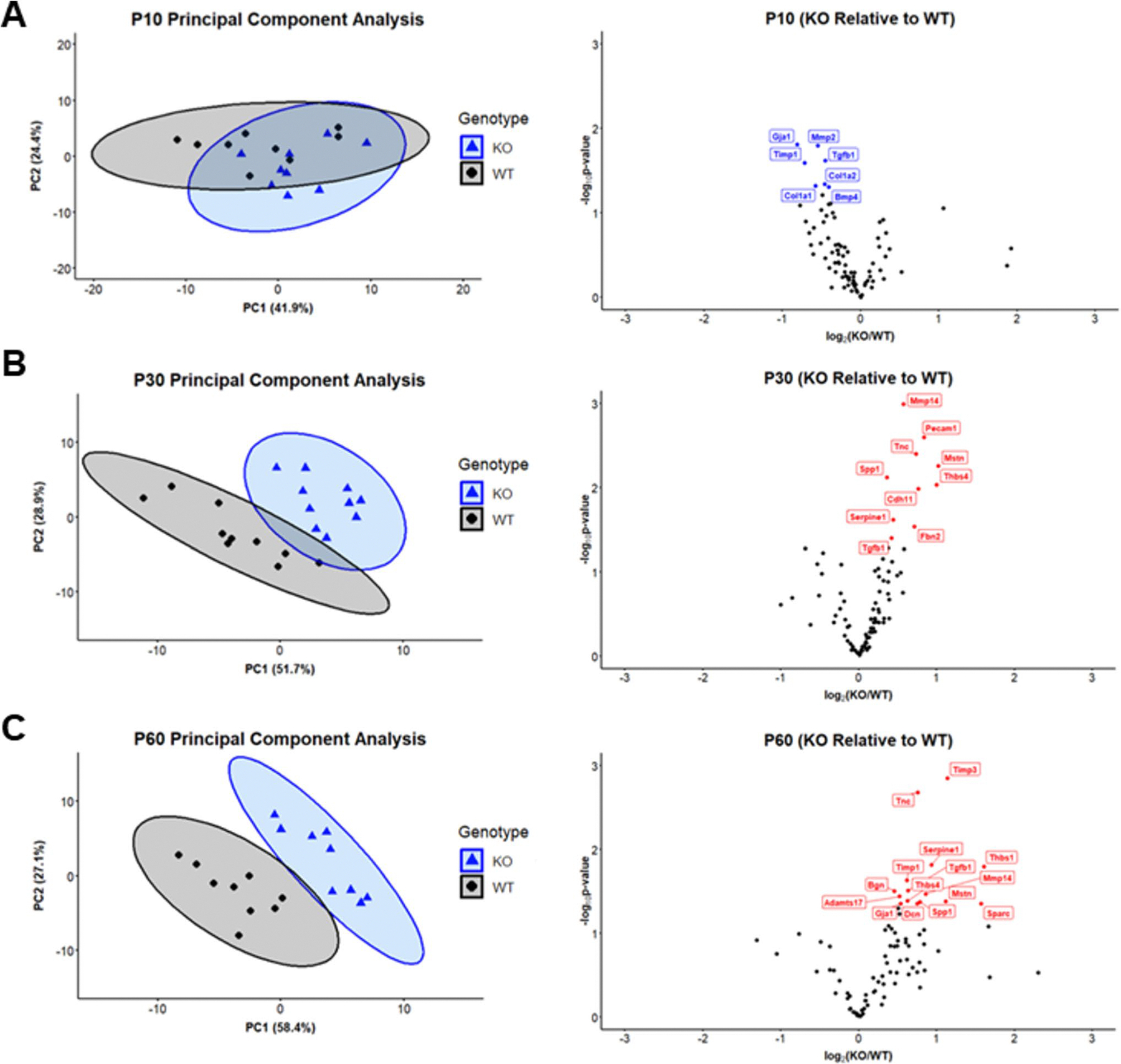
**A** P10 WT and KO tendons did not demonstrate distinct clustering via Principal Component Analysis. Genes such as *Gja1*, *Mmp2*, *Tgfb1*, *Timp1*, *Col1a1*, *Col1a2*, and *Bmp4* were downregulated in KO tendons relative to WT tendons. **B** P30 WT and KO tendons demonstrated distinct clustering via Principal Component Analysis. Genes such as *Mmp14*, *Pecam1*, *Tnc*, *Mstn*, *Spp1*, *Thbs4*, *Cdh11*, *Serpine1*, *Fbn2*, and *Tgfb1* were upregulated in KO tendons relative to WT tendons. **C** P60 WT and KO tendons demonstrated significant clustering via Principal Component Analysis. Genes such as *Timp3*, *Tnc*, *Serpine1*, *Thbs1*, *Thbs4*, *Timp1*, *Tgfb1*, *Mmp14*, *Bgn*, *Adamts17*, *Mstn*, *Sparc*, *Spp1*, *Dcn*, and *Gja1*

Gene expression was evaluated via high-throughput qPCR for 96 genes related to the classifications of collagens, non-collagenous matrix proteins, matrix remodeling, cell cycle markers, cell–cell and cell–ECM matrix interactions, and cell signaling pathways. We performed principal component analysis and used volcano plots to highlight the magnitude of differentially expressed genes in the KO tendons relative to WT tendons across all postnatal developmental timepoints. At P10, WT and KO tendons did not exhibit distinct clustering and volcano plot analysis revealed genes such as *Gja1*, *Mmp2*, *Tgfb1*, *Timp1*, *Col1a1*, *Col1a2*, and *Bmp4* were downregulated in KO tendons relative to WT tendons (Fig. 8A). At P30, principal component analysis revealed clustering between WT and KO tendons and volcano plot analysis revealed genes such as *Mmp14*, *Pecam1*, *Tnc*, *Mstn*, *Spp1*, *Thbs4*, *Cdh11*, *Serpine1*, *Fbn2*, and *Tgfb1* were upregulated in KO tendons relative to WT tendons. (Fig. 8B). At P60, principal component analysis revealed significant clustering between WT and KO tendons and genes such as *Timp3*, *Tnc*, *Serpine1*, *Thbs1*, *Thbs4*, *Timp1*, *Tgfb1*, *Mmp14*, *Bgn*, *Adamts17*, *Mstn*, *Sparc*, *Spp1*, *Dcn*, and *Gja1* were upregulated in KO tendons relative to WT tendons (Fig. 8C).

## Discussion

The objective of this study was to define the role of collagen XII in regulating the hierarchical assembly of the Achilles tendon required for function during postnatal development. Our approach involved tendon-targeted deletion of *Col12a1* using Scx-Cre;*Col12a1*^flox/flox^ mice, allowing us to examine how loss of collagen XII across the course of early postnatal development alters tendon structure, function, and gene expression. In accordance with our hypothesis, our findings demonstrate that collagen XII plays a key role in postnatal development of tendon, particularly at later timepoints and that its absence results in pronounced alterations in mechanical properties, collagen fibril structure, tendon length, cellular morphology, and gene expression.

One of the most consistent phenotypes across all postnatal timepoints was increased Achilles tendon length in KO mice. This phenotype was already evident at P10 and persisted through P60, suggesting impaired development may have been established prior to the onset of ambulation. While muscle loading can influence tendon morphogenesis and was not directly evaluated here, prior studies in both tendon-specific and global collagen XII knockout models have demonstrated muscle weakness [5, 16]. It is plausible that both reduced mechanical loading and intrinsic tendon abnormalities contribute to the phenotype [17, 18]. Additionally, since the Scx-Cre driver also exhibits expression in non-tendon tissues such as the growth plate and periosteum, alterations in insertion site development may also play a role in elongation [19, 20]. Future studies examining insertion site development and the contributions of muscle–tendon crosstalk in these mice could further clarify the mechanisms driving this phenotype.

Collagen XII deficiency may also contribute to increased tendon length through intrinsic, matrix-mediated mechanisms. Impaired fibril maturation and disrupted extracellular matrix organization may alter the mechanical integrity of the developing tendon, thereby influencing how load is transmitted during postnatal growth [4, 21, 22]. Collagen XII has been implicated in regulating cell–matrix interactions and mechanosensitive signaling, and disruption of these pathways may modify tenocyte behavior, including matrix deposition and alignment [4, 21]. Altered mechanotransductive cues during critical periods of postnatal development could therefore influence patterns of tendon growth and maturation [15, 23, 24]. Collectively, these matrix- and cell-mediated alterations may also have important implications for tendon mechanical function as development progresses.

Mechanical testing revealed progressive impairments in Achilles tendon function with collagen XII knockout. The observed decrease in dynamic modulus and increase in phase shift are indicative of reduced elastic behavior and greater energy dissipation [25]. These impairments suggest that collagen XII is necessary not only for static mechanical strength but also for dynamic mechanical competence under physiological loading conditions. The persistent nature of these alterations into P60, a timepoint corresponding with tendon maturation, underscores the long-term functional consequences of collagen XII deficiency. Additionally, impairments in collagen fiber realignment were most evident at P30 and P60, where KO tendons displayed greater normalized circular variance across 3–9% strain. This range encompasses the toe and linear regions where fiber realignment typically contributes to load accommodation [26]. These findings indicate that the collagen network in KO tendons is less capable of reorienting under tensile loading, likely due to disrupted interfibrillar organization. The observed mechanical deficits observed in KO tendons are consistent with a compromised extracellular matrix that lacks appropriate structural organization. This reduction in realignment could stem from compromised load transfer between fibrils and fibers, impairing the tendon’s ability to withstand mechanical stress under deformation, thus increasing the likelihood of tissue failure.

Differences between the mechanical property changes observed in this study and those reported in prior global *Col12a1* knockout studies likely reflect tendon-specific functional demands as well as differences in tissue specificity between models. In the global knockout model, FDL tendons exhibited increased cross-sectional area and stiffness [4], whereas Achilles tendons in the tendon-targeted knockout showed decreased stiffness without changes in cross-sectional area. These discrepancies may arise from intrinsic differences between positional tendons such as the flexor digitorum longus and energy-storing tendons, such as the Achilles, which experience distinct loading environments and rely on different structural mechanisms to support function [27, 28]. In addition, global knockout of collagen XII may produce secondary adaptations in muscle, bone, or other connective tissues that indirectly influence tendon structure and mechanics, whereas tendon-targeted deletion more directly isolates the tendon-autonomous role of collagen XII [4, 5].

Histological analyses revealed progressive changes in cell density and nuclear morphology in KO tendons. At P10, cell morphology was comparable between genotypes, but at P30 and P60, KO tendons exhibited increased cell density and reduced nuclear aspect ratio, indicative of more rounded nuclei. These findings suggest a loss of tenocyte polarity and spindle-shaped morphology, both hallmarks of mature tendon organization [29]. The disorganized cellular morphology may reflect impaired mechanotransduction or cytoskeletal tension, potentially contributing to transcriptional changes [15, 30].

Transmission electron microscopy confirmed disruptions in collagen fibrillogenesis. At P10, minor differences were observed between WT and KO fibril diameter distributions. Although this shift reached statistical significance, P10 represents an early developmental stage characterized by active cell proliferation and the transition toward extracellular matrix deposition [31]. At this stage, fibrillogenesis and lateral fibril growth are still ongoing, and the fibril diameter distributions exhibited substantial overlap between genotypes, suggesting limited functional relevance. However, by P30, KO tendons exhibited a notable shift toward smaller diameter fibrils, which became more pronounced at P60. These findings demonstrate that collagen XII may be required for normal lateral fibril growth during maturation. The accumulation of smaller fibrils and absence of large-diameter fibrils is consistent with prior studies of tendon pathology and suggests that collagen XII is involved with enabling the lateral fusion or expansion of collagen fibrils during matrix remodeling [4, 21, 32]. These ultrastructural abnormalities also suggest broader implications for tissue mechanics [33]. Smaller fibrils have a higher surface area-to-volume ratio and may be less efficient at bearing load compared to larger fibrils, which could directly contribute to the observed reductions in stiffness and modulus [34]. Moreover, aberrant fibrillogenesis may alter the biochemical composition of the matrix, as smaller fibrils can associate differently with proteoglycans and glycoproteins [35].

Gene expression analysis revealed distinct transcriptional signatures at each developmental timepoint. At P10, PCA showed limited separation between WT and KO groups, and volcano plots identified several downregulated genes including *Gja1*, *Mmp2*, *Tgfb1*, *Col1a1*, and *Col1a2* suggesting early matrix remodeling impairment. These genes are typically involved in gap junction formation, matrix degradation, and early matrix deposition, and their downregulation may reflect a lack of mechanical stimuli or altered matrix signaling in KO tendons [36–38]. Interestingly, connexin 43, a key protein in gap junctions and encoded by *Gja1*, is typically found at the contact points between adjacent cell processes [36]. However, in P14 tendons from *Col12a1* global knockout mice, connexin 43 appeared diffusely distributed rather than concentrated at cell junctions, indicating potential defects in intercellular communication [4]. Although this study did not assess connexin 43 localization directly, the downregulation of *Gja1* in KO tendons may suggest underlying alterations in both cell-cell and cell-matrix interactions in KO tendons. By P30 and P60, PCA revealed strong genotype-based clustering. At P30, upregulation of matrix remodeling and signaling genes such as *Mmp14*, *Mstn*, *Spp1*, and *Serpine1* was observed. At P60, KO tendons exhibited increased expression of protease inhibitors (*Timp1*, *Timp3*), matricellular proteins (*Tnc*, *Thbs1*, *Thbs4*), and regulators of matrix homeostasis (*Dcn*, *Adamts17*, *Sparc*). These changes point to a persistent remodeling response [39, 40]. Notably, TGF-β target genes (*Mstn*, *Serpine1*) were upregulated at both later timepoints despite no change in *Tgfb1*/*2*/*3*, suggesting increased TGF-β activity due to loss of sequestration by collagen XII [41, 42]. In other tissues, such as cornea and skin, collagen XII has been implicated in storing latent TGF-β within the matrix [21, 41]. Loss of this sequestration function may result in increased levels of bioavailable TGF-β, promoting excessive ECM production and fibrosis. These results support a model in which collagen XII functions as a modulator of TGF-β signaling during tendon development, and its absence disrupts the balance between synthesis and degradation in the ECM.

These findings support a model in which collagen XII regulates tendon development through multiscale mechanisms. Its absence disrupts early matrix assembly and tenocyte morphology and leads to persistent deficits in mechanical function and gene expression. The tendon appears to undergo a chronic remodeling response, but without appropriate structural templates, this remodeling is ineffective or maladaptive. Together, these data demonstrate that collagen XII is critical for coordinating the structural and molecular events required for proper development of postnatal tendon structure and function.

There are some limitations to the present study. First, the tendon-targeted *Col12a1* knockout model, generated using a scleraxis-Cre driver, was chosen to avoid the perinatal lethality associated with global knockouts and to minimize systemic off-target effects that could influence tendon outcomes. However, because scleraxis is also expressed in muscle and bone cells [43, 44], these tissues may have contributed to some of the observed findings, particularly tendon elongation and rupture. Future studies could more precisely isolate the tendon-specific roles of collagen XII by employing alternative tendon-specific Cre drivers, such as tenomodulin (*Tnmd*)-Cre. In addition, tissue-specific rescue experiments, in which collagen XII expression is restored selectively within tendon tissue, could be used to verify whether the observed phenotypes arise from tendon-autonomous effects rather than secondary contributions from surrounding musculoskeletal tissues. In addition, collagen XII deficiency produced pronounced changes in mechanical properties along with a large number of differentially expressed genes. Gene expression analysis in this study was performed using a targeted, hypothesis-driven qPCR panel composed of pre-selected genes related to extracellular matrix organization, matrix remodeling, cell-matrix interactions, and mechanosensitive signaling pathways relevant to tendon development. Because this gene set is not genome wide and is already functionally enriched by design, pathway enrichment analyses such as GO or KEGG would be statistically constrained and potentially misleading and were therefore not performed. This represents a limitation of the present study. Instead, interpretation focused on linking stage-specific transcriptional changes to biological processes involved in tendon maturation, matrix assembly, and remodeling. Future transcriptome-wide approaches may build upon these findings to identify broader signaling networks regulated by collagen XII during tendon development. Since transcriptional changes do not always reflect protein-level differences, further work is needed to validate whether compositional changes underlie the mechanical alterations. A related limitation is the lack of direct matrix compositional analysis. Although key matrix genes were differentially expressed, it remains unclear whether these changes correspond to altered protein abundance. Future investigations using proteomic approaches, such as nano-flow liquid chromatography tandem mass spectrometry, could address this gap by examining age-related differences in protein composition [45]. Finally, tendon behavior can vary depending on functional demands [46–48], and it is possible that tendons other than those studied here may respond differently to the loss of collagen XII.

Looking ahead, several open questions remain. It is unclear whether the observed transcriptional changes are a direct response to altered mechanotransduction, changes in nuclear morphology, or indirect effects of disrupted ECM architecture. Collagen XII has been shown to be a mechanosensitive collagen [21, 49, 50] and future studies may aim to understand the role of collagen XII with altered mechanical loading in development of hierarchical tendon structure and function. Additionally, future studies may also incorporate single-cell transcriptomics or in situ hybridization techniques to understand how specific cell populations regulate the expression of collagen XII and how its spatial distribution correlates with potential site-specific structure-function within the Achilles tendon [21]. Additionally, the specific roles of the short and long isoforms of collagen XII [51] remain to be determined. Isoform-specific knockout models or overexpression systems could help delineate whether structural versus regulatory functions of collagen XII are differentially mediated. Finally, studies involving targeted therapeutic strategies aimed at modulating TGF-β signaling or reinforcing matrix integrity could hold promise for addressing connective tissue dysfunction in collagen XII-deficient contexts, including myopathic Ehlers-Danlos Syndrome.

Together, these findings advance understanding of how hierarchical extracellular matrix organization is established during postnatal tendon development and how disruption of collagen XII alters multiscale structure-function and gene expression. By defining a tendon-specific role for collagen XII, this work provides insight into how matrix-mediated signaling, fibrillogenesis, and mechanotransduction are integrated during tendon development and maturation. These findings have implications for congenital connective tissue disorders, including myopathic Ehlers–Danlos syndrome, and establish a framework for future studies aimed at identifying therapeutic strategies to promote functional tendon development and repair.

The results from this study demonstrated that collagen XII is a critical regulator of tendon length, mechanical function, fibril organization, cellular architecture, and ECM remodeling during later stages of postnatal development. Loss of collagen XII results in persistent and multifaceted impairments that span molecular, structural, and functional levels. These findings support a comprehensive model in which collagen XII integrates cell–matrix and matrix–matrix interactions, mechanical signaling, and fibrillogenesis to establish proper development of tendon hierarchical structure and mechanical function.

## Supplementary Material

Suppl 1

Suppl 2

**Supplementary Information** The online version contains supplementary material available at https://doi.org/10.1007/s10439-026-04125-6.
